# Adsorption of Arsenic by Magnetically Modified Biochar from Mulberry Tree Stems

**DOI:** 10.3390/toxics13110951

**Published:** 2025-11-04

**Authors:** Sheng Hu, Jiayu Yang, Jingnan Zhang, Jing Pan, Liling Yan, Kun Dong, Dunqiu Wang, Ying Song, Meina Liang

**Affiliations:** 1 Guangxi Key Laboratory of Environmental Pollution Control Theory and Technology, Guilin University of Technology, Guilin 541004, China; 18516911386@163.com (S.H.); yangjiayu@glut.edu.cn (J.Y.); zhangjingnan9@163.com (J.Z.); 15296239879@163.com (J.P.); yanlilingg@163.com (L.Y.); 2020005@glut.edu.cn (K.D.); wangdunqiu@glut.edu.cn (D.W.); 2009005@glut.edu.cn (Y.S.); 2 Engineering Research Center of Watershed Protection and Green Development, Guilin University of Technology, Guilin 541006, China; 3 Guangxi Engineering Research Center of Comprehensive Treatment for Agricultural Non-Point Source Pollution, Guilin University of Technology, Guilin 541006, China; 4 Modern Industry College of Ecology and Environmental Protection, Guilin University of Technology, Guilin 541006, China; 5 Collaborative Innovation Center for Water Pollution Control and Water Safety in Karst Area, Guilin University of Technology, Guilin 541006, China; 6 University of Chinese Academy of Social Sciences, Beijing 102488, China

**Keywords:** mulberry stem biochar, adsorbent, As, magnetic modification

## Abstract

In this study, mulberry tree stems were used as raw material to prepare magnetic modified biochar, Fe-BC-500, using the co-precipitation method. The structure of Fe-BC-500 was systematically characterized and tested for arsenic (As) adsorption in batch experiments by varying parameters such as solution pH (3–11), the concentrations of co-existing anions (2–20 mg/L), and ionic strength (0–0.5 mol/L NaNO_3_). The results indicate that Fe-BC-500 exhibited optimal adsorption capacity at pH 4 and an initial As(V) concentration of 20 mg/L. The influence of co-existing anions on As(V) adsorption followed the order PO_4_^3−^ > SO4^2−^ > NO_3_^−^. Kinetic analysis showed that adsorption of Fe-BC-500 on As(V) followed a pseudo-second-order kinetic model, with a correlation coefficient of 1.00, indicating chemical adsorption. The Langmuir model accurately described the isothermal adsorption results, indicating monolayer adsorption. Mechanistic analysis showed that As(V) was fixed on the Fe-BC-500 surface through complexation reactions, demonstrating adsorption specificity. This study provides a theoretical basis and highlights the application potential of magnetically modified biochar for removing As(V) from water.

## 1. Introduction

Arsenic (As) is a metalloid with intermittent properties of both metals and non-metals. As exists in four different valence states, −3, 0, +3, and +5, and is considered one of the most dangerous elements [1]. As is typically characterized by high solubility and significant toxicity at low concentrations [2]. In natural environments, it mainly exists in association with various metal ores, such as Cu and Pb, in the form of chalcopyrite (i.e., As_2_S_3_ for orpiment, As_4_S_4_ for realgar, and FeAsS for arsenopyrite). These minerals are often used industrially as pesticides, herbicides, insecticides, or alloys. China produces over 30,000 Mg of As waste annually via the mining of Cu, Pb, and Zn ores [3]. The As content in natural environments is relatively low, with concentrations in soil and rocks generally ranging from 1 to 20 and 0.5 to 2.5 mg/kg, respectively; these concentrations do not harm human health [4]. As in solution mainly exists in the form of trivalent As [As(III)] and pentavalent As [As(V)] [5], among which monomethyl arsenate, dimethyl arsenate, and trimethyl arsenate are considered the main toxic forms in natural water. As concentrations in water that exceed a certain level can cause toxicity in both animals and humans [6,7]. As(V) can be reduced to As(III) in human cells, causing skin diseases and cancer [8]. Arsenic pollution control is an important environmental issue at both the global and national levels. The World Health Organization has set the maximum concentration of As in drinking water to 10 μg/L; the As concentration in natural water is 0.001–10 μg/L [9,10,11]. Following this international benchmark, China has formulated a national policy through the Ministry of Ecology and Environment to strengthen the remediation and control of pollution by heavy metals such as As. This commitment is specifically reflected in the PRC National Standard, GB3838-2002 [12], which clearly stipulates that the As content limit for Class III surface waters is 0.05 mg/L (surface water-Class III As control index) [13]. The implementation of these policies has profound implications for safeguarding public health, achieving sustainable economic development, and promoting sustainable co-existence between humans and nature. It can lead to a variety of diseases, such as cardiovascular diseases, hyperkeratosis of the palms and soles, central nervous system damage, liver damage, hair loss, skin cancer, and visceral cancer (lung cancer, liver, kidney, bladder cancer) [14].

High As pollution in the environment is primarily due to human activity [15]. Human activities such as mining [16], smelting [17], application of As-containing pesticides and fertilizers [18], preservation of wood, discharge of industrial wastewater, and agricultural irrigation have caused varying degrees of As pollution in soils worldwide. According to a survey, tens of thousands of As pollution sites exist worldwide, with the highest soil As content reported at 250,000 mg/kg [19]. In areas such as Kuitun in Xinjiang, Datong in Shanxi, Yuncheng in Shanxi, Hetao in Inner Mongolia, Yinchuan in Gansu, and the Songnen Plain in Northeast China, high-As groundwater irrigation has substantially polluted farmland [20]. Hunan, Hubei, Guangxi, Yunnan, and other provinces have also experienced moderate or severe As pollution in soil due to mining and smelting activities. As is widely present in soil and is difficult to degrade, causes persistent harm, and accumulates in the food chain. Because it exerts various negative effects on human health [21], the public is becoming increasingly concerned about the accumulation of metals and metalloid As in agricultural soils [22]. As can further penetrate groundwater and water bodies, causing damage to aquatic ecosystems and toxic effects on fish and other aquatic organisms [23]. There are numerous reports on As pollution in water, both domestically and internationally. In the Santurb’an Paro region of Colombia, the mining industry has expanded the As contaminated area [24]. In southeastern Bangladesh, the excessive exploitation of groundwater has led to a decrease in groundwater levels and changes in organic matter, resulting in the release of As and accumulation in aquifer systems [25]. In the Yinchuan Plain in China, the infiltration of agricultural irrigation water led to an increase in As concentrations in groundwater [26]. In China, high-As groundwater is widely distributed in more than 20 provinces, with Xinjiang, Shanxi, Inner Mongolia, Jilin, and Ningxia being the most severely affected provinces [27,28]. Therefore, remediation of As pollution is urgently needed.

Owing to the increasingly severe environmental problems associated with As pollution, various technical methods for repairing As pollution in water bodies have been developed. The primary removal methods include precipitation, ion exchange, membrane separation, adsorption, electrolysis, and biological methods [29,30,31]. Based on the characteristics and geographical locations of polluted water sources, different removal technologies are often combined to achieve optimal restoration. Among these, adsorption has become one of the most widely used technologies for As treatment in water because of its high removal efficiency, low cost, and ease of operation. The adsorption method mainly uses porous solid materials to adsorb As ions from water through physicochemical reactions, achieving separation of As and water [32]. A variety of adsorbents have been employed for the removal of As from water, including activated carbon, granular ferric hydroxide, magnetic graphene oxide, iron oxide, zeolite, nanocomposites, ion exchange resins, and biochar [33].

Biochar is a carbon-rich solid material produced by the pyrolysis of biomass under anaerobic or oxygen-limited conditions (International Biochar Initiative, IBI) [34]. The feedstocks used include wood, leaves, straw, nut shells, animal manure, municipal sludge, and others [35,36]. The production of biochar is low-cost and environmental-friendly, and biochar has often been used for the remediation of As-containing wastewater [36,37,38]. Biochar can adsorb and fix heavy metals in soil through electrostatic adsorption, ion exchange, co-precipitation, and complexation of oxygen functional groups and π electrons [39]. Conventional biochar also has some limitations such as limited adsorption capacity and a narrow adsorption range [40]. Therefore, many researchers have aimed at producing modified biochar with a stronger adsorption capacity to enhance its adsorption effect. Zeng et al. [41] impregnated rice straw with manganese chloride and then pyrolyzed it to produce manganese modified biochar. The removal rate of As by this biochar was 86%, and the removal equilibrium was reached within 48 h. These materials had remarkable catalytic oxidation capability, converting over 90% of As(III) to As(V) upon addition of H_2_O_2_. Nguyen et al. [42] successfully prepared a novel bimetallic zinc–iron-modified biochar using sugarcane bagasse. This biochar had significantly improved adsorption performance for As(III) in water through the synergistic effect of zinc and iron. Adsorption experiments showed that the maximum adsorption capacity for As(III) was 34.7 mg·g^−1^, indicating that the material has important application value in the remediation of As-containing wastewater. Gregory et al. [43] found that willow biochar was beneficial for the remediation of As-contaminated soil. Bano et al. [44] modified chestnut shells with magnetic gelatin and found an increased adsorption capacity (by 28.30 mg/g) of biochar produced from these shells compared with that of biochar produced from unmodified shells.

In this study, we used a co-precipitation method to prepare magnetically modified biochar, Fe-BC-500, from mulberry stems. The effects of Fe-BC-500 on the adsorption of As(V) in wastewater at different pH values, initial concentrations of As in solution, co-existing anions (SO_4_^2−^, NO_3_^−^, and PO_4_^3−^), and ion strengths (NO_3_^−^) were investigated. The data were analyzed by establishing an adsorption kinetics model and isotherm fitting. We constructed a magnetically modified biochar material system that provides information on material preparation and mechanistic evidence for potential engineering applications. We combined X-ray diffraction (XRD), Fourier-transform infrared (FTIR) spectrometry, and scanning-electron microscopy (SEM) analyses to evaluate the properties of FE-BC-500 before and after adsorption of As(V). We systematically assessed the potential of FE-BC-500 in efficiently removing As(V) from wastewater, thus laying a theoretical and experimental foundation for the application of As remediation technologies in water bodies. We further provide a theoretical basis for the utilization of mulberry stem biochar and explore prospects of the re-utilization of agricultural waste.

## 2. Materials and Methods

### 2.1. Experimental Materials

The mulberry stems used in this study were obtained from Huanjiang County, Hechi City, Guangxi Province, China. After peeling the stems, they were dried at 105 °C, crushed with a grinder, and sieved through a 20-mesh sieve for later use [45]. The main experimental reagents included analytical-grade C_6_H_8_O_6_, CH_4_N_2_S, Na_3_AsO_4_·12H_2_O, Na_2_SO_4_, NaNO_3_, and Na_3_PO_4_, and high-grade KBH_4_ and KOH, all of which were sourced from Xilong Chemical Co., Ltd., Shantou, China. The As standard solution was obtained from the National Steel Materials Testing Center Iron and Steel Research Institute, and pure spectral KBr was obtained from Nanjing Chemical Reagent Co., Ltd., Nanjing, China.

### 2.2. Preparation of Mulberry Stem Materials

**Figure 1:**
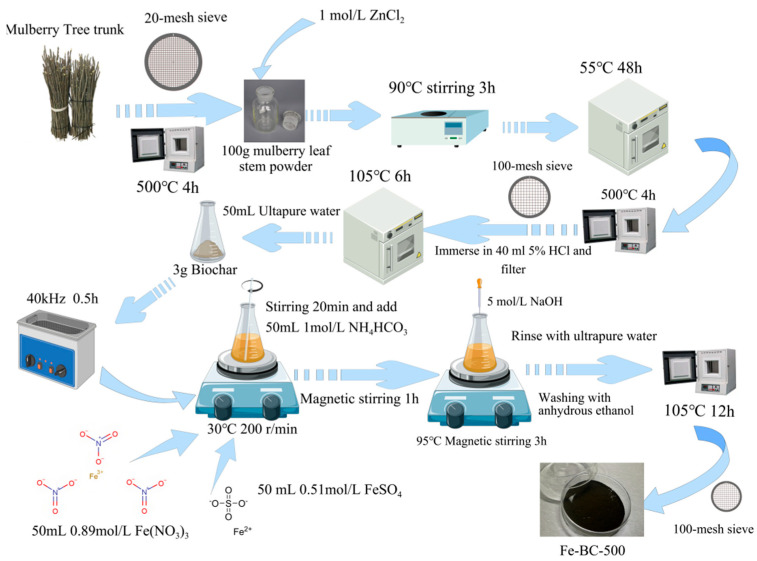
Effect of pH on As(V) adsorption onto Fe-BC-500.

We mixed 100 g of mulberry stem powder sieved through a 20-mesh sieve with 1 mol/L ZnCl_2_ solution in a 2.5 L wide mouthed bottle, stirred the mixture in a 90 °C water bath for 3 h, and filtered and dried the solution at 55 °C for 48 h. The dried sample was carbonized in a muffle furnace at 500 °C for 4 h, ground through a 100-mesh sieve, and soaked in 5% HCl solution. The solution was then washed until attaining neutral pH, filtered, and dried at 105 °C for 6 h. Three grams of the obtained biochar was transferred to a conical flask, 50 mL of ultrapure water was added, and the mixture was sonicated for 0.5 h. Then, 50 mL of Fe(NO_3_)_3_/FeSO_4_ solution was added at 30 °C and the mixture stirred for 20 min. Fifty milliliters of 1 mol/L NH_4_HCO_3_ was then added and stirring continued. After 1 h, the pH was adjusted to 10.5 with NaOH and the solution heated at 95 °C for 3 h. After cooling, the solution was filtered, washed with water until neutral pH was attained, washed with ethanol once, dried at 105 °C for 12 h, and passed through a 100-mesh sieve. Magnetic iron oxide/mulberry stem biochar was thus obtained. Unmodified biochar carbonized at 500 °C is referred to as M-BC-500, while the magnetically modified biochar is referred to as Fe-BC-500 [46]. A flowchart of the material preparation is shown in Figure 1.

### 2.3. Characterization Methods

#### 2.3.1. FTIR Analysis (FTIR Spectroscopy Testing and Baseline Correction)

The samples were thoroughly mixed with KBr in an agate grinding bowl at a mass ratio of 1:80. The mixture was then placed in a 13 mm KBr thick plate mold and pressed into a uniform transmission plate. The pressing time was 1 min, which ensured that the surface was flat and free of cracks. Measurement in transmission mode was conducted using a Nissan Nicolet 6700 FTIR instrument, which were sourced from Thermo Fisher Scientific, Fitchburg, WI, USA. With a spectral band coverage of 500–4000 cm^−1^ and a resolution of 4 cm^−1^. Each sample was scanned 32 times; the background was scanned 64 times under the same conditions and subtracted. To reduce moisture interference, KBr and the sample were kept as dry as possible; the operating environment was also kept dry. OMNIC 9.xwas used for background subtraction, baseline correction, and smoothing and normalization. Finally, key peak positions were determined and assigned to functional groups based on a standard spectral library [47].

#### 2.3.2. X-Ray Diffraction Analysis (XRD Experimental Design and Data Acquisition)

The samples were analyzed using a Bruker-axs D8 ADVANCE X-ray diffractometer [48], which were sourced from Bruker Corporation, Billerica, MA, USA. With Cu target Kα radiation and a characteristic X-ray wavelength of λ = 1.54059°. The diffraction angle range was 2θ = 10–90°, and the diffraction rate was 4°/min. Appropriate steps (0.02°) and counting times for each step (0.5–2 s) were combined to obtain regular peak shapes and statistical signals. The samples were placed on a flat sample holder and rotated and aligned as necessary to improve repeatability. To ensure the comparability of results, the instrument was calibrated with standard samples to confirm the 2θ zero point, peak position resolution, and resolution specificity.

#### 2.3.3. Scanning Electron Microscopy (SEM Sample Preparation and Imaging Parameter Configuration)

After gold plating treatment, the samples were fixed on the SEM-sample stage, and conductive tape or silver paste was used to ensure sufficient grounding between the sample, sample stage, and cavity. Subsequently, the sample stage was placed in the high-vacuum sample chamber of a JEOL JSM-7900F field-emission scanning electron microscope, which were sourced from Japan Electron Optics Laboratory Co., Ltd., Tokyo, Japan. After generating sufficient vacuum, the morphological characteristics of Fe-BC-500 before and after adsorption of As(V) were observed. The sample preparation process was as follows: The sample was uniformly attached to a sample stage covered with a conductive adhesive. After gold plating, microscopic observation was performed under an acceleration voltage of 3–4 kV. Representative microscopic images were captured for each sample at a magnification of 10.00k× [49].

### 2.4. Adsorption Experiment

#### 2.4.1. Influence of Solution pH on Adsorption Efficiency

We transferred 0.1 g of Fe-BC-500 to a 100 mL plastic centrifuge tube. Then, 50 mL of an As(V)-containing solution, whose pH was adjusted to a range of 3.0–11.0 using 0.1 mol/L NaOH or HCl solution, was added. After thorough shaking, the pH was adjusted to the target value using the same procedure. The mixture was oscillated at 25 °C and 200 rpm for 60 h to allow adsorption. After the adsorption reaction was complete, the plastic centrifuge tube was removed and centrifuged at 4000 r/min for 10 min. The supernatant was drawn with a syringe, filtered through a 0.22-µm membrane, and the concentration of residual As(V) in the filtrate was analyzed using atomic fluorescence spectrometry [50].

#### 2.4.2. Influence of Co-Existing Ions on Adsorption Efficiency

A 0.1 g sample of Fe-BC-500 was placed in a 100 mL plastic centrifuge tube. Then, 50 mL of As(V) solution containing SO_4_^2−^, NO_3_^−^, and PO_4_^3−^ at three concentrations (2, 10, and 20 mg/L) was added. The pH of the mixture was adjusted to 4 using 0.1 mol/L NaOH or HCl, followed by thorough shaking. This pH adjustment procedure was repeated twice to ensure the stability of the target value. These experiments were designed to investigate the effects of co-existing ions on the adsorption of As(V) by Fe-BC-500 [51].

#### 2.4.3. Influence of Ionic Strength on Adsorption Efficiency

A 0.1 g sample of Fe-BC-500 was placed in a 100 mL plastic centrifuge tube. Then, 50 mL of an As(V) solution containing 0.05–0.5 mol/L NaNO_3_ was added. The pH of the mixture was adjusted to 4 following the procedure described in Section 2.4.1, and an adsorption experiment was conducted. After adsorption, the residual As(V) concentration was determined according to the analytical protocol outlined in Section.

#### 2.4.4. Adsorption Kinetics Experiments

A 0.1 g sample of Fe-BC-500 was placed in a 100 mL plastic centrifuge tube. Subsequently, 50 mL of As(V) solution with initial concentrations of 10 and 20 mg/L was added. The pH of the mixture was adjusted to 4 using 0.1 mol/L NaOH or HCl, followed by thorough shaking. This pH adjustment procedure was repeated twice to ensure the stability of the target value. The adsorption experiment was performed at 25 °C with shaking at 200 r/min in a constant-temperature water bath. Samples were collected at different time intervals (0.5, 1, 1.5, 3, 6, 9, 12, 18, 24, 30, 36, 42, 48, 60, 72, 84, 96 h). After the adsorption reaction was complete, the tube was removed and centrifuged at 4000 rpm for 10 min. The supernatant was then filtered and the residual As(V) concentration was analyzed using atomic fluorescence spectroscopy. Calculations were performed using Equations (1) and (2) [52,53].

(1) $$\ln(Q_{e}-Q_{t})=\ln Q_{e}-\frac{K_{1}t}{2.303}$$

(2) $$\frac{t}{Q_{t}}=\frac{t}{Q_{e}}+\frac{1}{K_{2}{Q_{e}}^{2}}$$

where t is the reaction time, min; Q_t_ is the adsorption capacity at time t, mg/g; K_1_ is the rate constant of the first-order kinetic model, min^−1^; and K_2_ is the rate constant of the second-order kinetic model, g/(mg·min).

The equilibrium adsorption capacity (Q_e_) and removal rate (R_e_) of As(V) in solution were calculated as shown in Equations (3) and (4) [54,55].

(3) $$Q_{e}=\frac{(C_{0}-C_{t})\times V}{m}$$

(4) $$R_{e}=\frac{(C_{0}-C_{t})\times 100\%}{C_{0}}$$

where C_0_ and C_t_ are the initial and final concentrations of the As(V) solution, respectively (mg/L); V is the volume of the solution (L); and m is the amount of adsorbent added (mg).

##### Bangham Dynamics

The Bangham Dynamics model describes the adsorption kinetics of porous solid adsorbents for gases or liquids and is commonly employed to analyze the early stages of adsorption, in which adsorption rates are governed by intraparticle diffusion [56].

The equation used is as follows:

(5) $$Q_{t}=K_{3}\cdot t^{1/n}$$

where t is the reaction time, min; Q_t_ is the adsorption capacity at time t, mg/g; K_3_ is the Bangham kinetic constant; and *n* is an empirical constant, typically ranging between 1 and 3.

##### Elovich Dynamics

The Elovich kinetic equation is commonly used for describing time-dependent adsorption rates during chemical adsorption processes. This equation is particularly suited for situations in which the adsorption rate decreases exponentially with increasing surface coverage [57].

The equation used is as follows:

(6) $$Q_{t}=K_{4}t^{1/2}+c$$

where t is the reaction time (min), Q_t_ is the adsorption capacity at time t (mg/g), and K_4_ is the Elovich kinetics constant.

## 3. Results and Discussion

### 3.1. Effect of Adsorption of As(V) on Fe-BC-500 at Different pH Values

**Figure 2:**
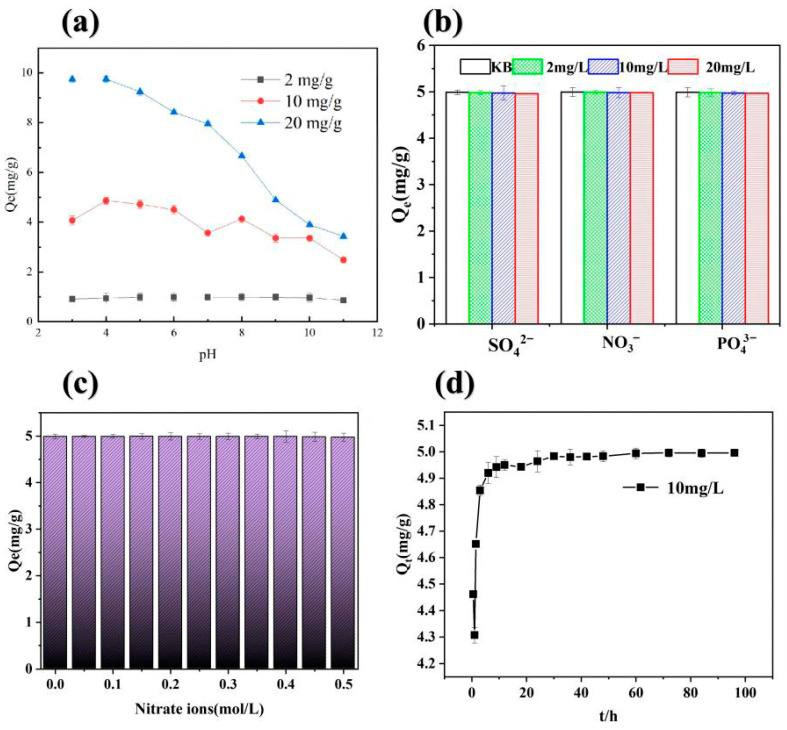
Effect factors on As(V) adsorption on Fe-BC-500 (**a**) Effect of pH on As(V) adsorption on Fe-BC-500; (**b**) Effects of co-existing anions on As(V) adsorption on Fe-BC-500; (**c**) Effect of ionic strength on As(V) adsorption on Fe-BC-500; (**d**) Effect of contact time on As(V) adsorption on Fe-BC-500.

The adsorption performance of Fe-BC-500 for As(V) under different pH conditions is shown in Figure 2a (initial concentration: 2 mg/L). The results showed that changes in solution pH had no significant effect on the adsorption capacity (Q_e_). This result is in line with that of Devrajani et al. [58], who found no variations in the As adsorption capacity of brown seaweed iron-modified biochar based on pH. This is mainly because, at this low concentration, the adsorbent surface has sufficient active sites to maintain the removal rate of As(V) at a high level. Therefore, at low As(V) concentrations, the influence of pH is limited. Statistical analysis revealed a significant positive correlation between the adsorption capacities at 10 and 20 mg/g (Pearson’s r = 0.864, *p* < 0.01, *n* = 11). In contrast, the capacities at 2 mg/g were not significantly correlated with those at 10 mg/g (r = 0.531, *p* > 0.05, *n* = 11) or 20 mg/g (r = 0.312, *p* > 0.05, *n* = 11), indicating a strong linear relationship only within a higher adsorption range. At As(V) concentrations of 10 and 20 mg/L, the effect of solution pH on As(V) removal from water gradually increased. When the pH increased from 3.0 to 4.0, the adsorption capacity also increased. When the pH was between 4.0 and 7.0, the As(V) adsorption capacity decreased faster in the 20 mg/L solution than in the 10 mg/L solution. At pH values above 7, the adsorption capacity of Fe-BC-500 for As(V) in water gradually decreased as the pH increased. Fe-BC-500 exhibited the highest adsorption capacity and best adsorption effect at pH 4. The removal rates at different concentrations (2, 10, and 20 mg/L) were 99.1, 99.7, and 97.5%, respectively, which were 1.1, 2.0, and 2.9 times the corresponding adsorption capacities at pH 11. These differences arose because the pH of the solution affects the form of As in water and the surface charge of the adsorbent. As(V) in solution exists in different forms at different pH values. At pH values below 2.2, As(V) mainly exists in the form of H_3_AsO_4_ [59]; at pH values between 2 and 7, As(V) mainly exists in the form of H_2_AsO_4_^−^ [60]; at pH values between 7 and 11, As(V) mainly exists as HAsO_4_^2−^ [61]; and at pH values above 11, As(V) mainly exists as AsO_4_^3−^ [62]. The zero-point of charge of Fe-BC-500 was at pH_zpc_ = 8.74. At pH values between 3 and 8.74, the Fe-BC-500 surface carries a positive charge, and As(V) in solution mainly exists as H_2_AsO_4_^−^ and HAsO_4_^2−^. Under these conditions, Fe-BC-500 electrostatically attracts As(V), which is conducive to the adsorption of anions. At pH values between 8.74 and 11, the surface of Fe-BC-500 is negatively charged and As(V) mainly exists as H_2_AsO_4_^−^. Under these conditions, the adsorbent and adsorbate electrostatically repel each other, which is not conducive to the adsorption of As(V) on Fe-BC-500. Therefore, as the pH increased, the adsorption capacity of Fe-BC-500 gradually decreased.

### 3.2. Influence of Co-Existing Ions on As(V) Adsorption on Fe-BC-500

In addition to As(V) ions, different co-existing anions exist in wastewater that can compete for adsorption sites on the adsorbent surface, thereby affecting adsorption efficiency. We selected Na_2_SO_4_, NaNO_3_, and Na_3_PO_4_ as co-existing anions of SO_4_^2−^, NO_3_^−^, and PO_4_^3−^. We added 0.1 g of Fe-BC-500 to 50 mL of 10 mg/L As(V) solution at pH 4 and a temperature of 25 °C to study the effects of the selected anions on As(V) adsorption on Fe-BC-500. The experimental results are shown in Figure 2b. The Q_e_ values of the KB, 2-, 10-, and 20 mg/L groups for SO_4_^2^ ions were 4.992, 4.982, 4.973, and 4.961 mg/g, respectively. The Q_e_ values of the KB, 2, 10, and 20 mg/L-groups for NO_3_^−^ and PO_4_^3−^ ions were 4.993, 4.993, 4.982, and 4.981 mg/g, and 4.992, 4.983, 4.971, and 4.969 mg/g, respectively. As shown in Figure 2b, the effects of SO_4_^2−^, NO_3_^−^, and PO_4_^3−^ on the adsorption of As(V) on Fe-BC-500 were non-significant. The addition of 2 mg/L NO_3_^−^ to the solution had no effect on the adsorption of As(V) on Fe-BC-500. At NO_3_^−^ concentrations of 10 mg/L, the adsorption of As(V) on Fe-BC-500 decreased by 0.01 mg/g (0.2%). For the 10 mg/L PO_4_^3−^ solution, the adsorption of As(V) on Fe-BC-500 in the KB group decreased by 0.02 mg/g (0.4%), a reduction twice as high as that induced by NO_3_^−^ ions. Although PO_4_^3−^ and As(V) compete for adsorption sites, the ability of As(V) to bind to iron oxides is stronger than that of PO_4_^3−^ ions. The impact of different concentrations of SO_4_^2−^, NO_3_^−^, and PO_4_^3−^ on the adsorption of As(V) varied in the order PO_4_^3−^ > SO_4_^2−^ > NO_3_^−^, which is consistent with the results of Islam et al. [63].

### 3.3. As(V) Adsorption on Fe-BC-500 at Different Ionic Strengths

The effect of different concentrations of NO_3_^−^ ions (0, 0.05, 0.1, 0.15, 0.2, 0.25, 0.3, 0.35, 0.4, 0.45, 0.5, 1 mol/L) on the adsorption of As(V) on Fe-BC-500 was studied at pH 4 and a reaction temperature of 25 °C. The experimental results are shown in Figure 2c. The effects of different NO_3_^−^ concentrations on As(V) removal by Fe-BC-500 were non-significant. As the NO_3_^−^ ion strength increased from 0.05 to 0.50 mol/L, there was a slight decreasing trend. At a NO_3_^−^ concentration in the solution between 0.05 and 0.35 mol/L, the adsorption capacity of Fe-BC-500 for As(V) remained unaltered. At increased concentrations of 0.4 and 0.5 mol/L, the adsorption capacity of Fe-BC-500 decreased by 0.19 and 0.38%, respectively. The effect of NO_3_^−^ on the removal of As(V) by Fe-BC-500 was non-significant, indicating low competition between As(V) and NO_3_^−^ for Fe-BC-500 adsorption sites. During adsorption, As(V) forms a complex on the inner surface of Fe-BC-500 [64]. At pH 4, As(V) mainly exists as H_2_AsO_4_^−^ and can form chemical bonds such as Fe-O-As with Fe-O or Fe-OH surfaces, which represents strong “chemical bonding” rather than electrostatic adsorption. Moreover, the Fe-BC-500 surface is positively charged at pH 4, which enables strong binding with the negatively charged As(V). These bonds are difficult to displace by NO_3_^−^. The inner composite layer of Fe-BC-500 is not as strongly affected by the background ion strength as the outer composite layer; therefore, it can still maintain high activity at higher NO_3_^−^ concentrations.

### 3.4. Influence of Different Adsorption Times on Adsorption of As(V) on Fe-BC-500

We tested the effect of different adsorption times (0.5, 1, 1.5, 3, 6, 9, 12, 18, 24, 30, 36, 42, 48, 60, 72, 84 and 96 h) for 0.1 g of Fe-BC-500 adsorbent added to 50 mL of As(V) wastewater with an initial concentration of 10 mg/L. The reaction was conducted at a temperature of 25 °C, and the results are shown in Figure 2d. The adsorption capacity of Fe-BC-500 for As(V) gradually increases with increasing contact time. In the first 9 h, the adsorption reaction rate was relatively high [0.55 mg/(g·h)] because the adsorbent surface contained numerous absorption sites in the initial stage. Over time, the adsorption curve gradually became smoother, and the adsorption reaction rate decreased. This may have been due to the decreasing number of adsorption sites on the surface of Fe-BC-500. This observation was consistent with the findings of Lipton et al. [65], who found that the optimal adsorption time for As removal was 90 min. The adsorbed As(V) gradually diffused inward from the surface, the adsorption rate decreased, and adsorption equilibrium was reached at 60 h.

### 3.5. Adsorption Kinetics Model

**Figure 3:**
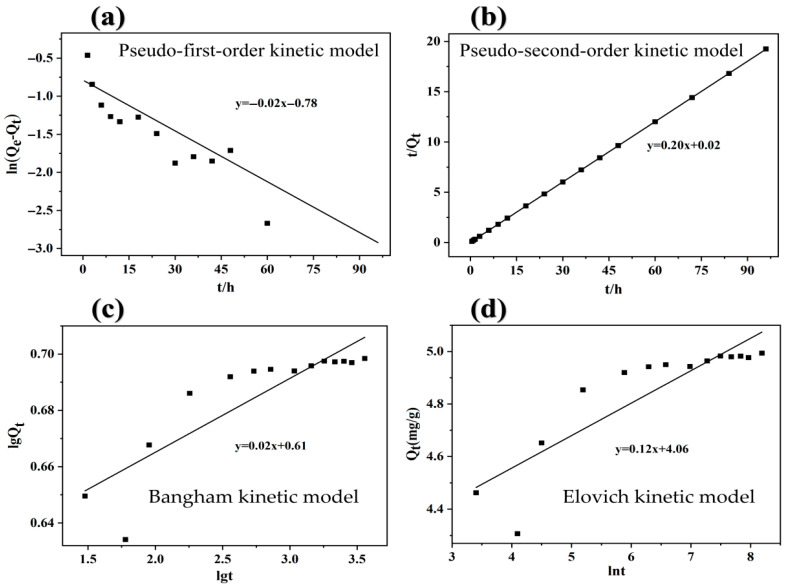
(**a**) Pseudo-first-order kinetic model; (**b**) Pseudo-second-order kinetic model; (**c**) Bangham kinetic model; (**d**) Elovich kinetic model.

The experimental adsorption data were fitted using pseudo-first-order, pseudo-second-order, Bangham, and Elovich kinetics, shown in Figure 3. The fitting parameters for the adsorption kinetics are listed in Table 1. The pseudo-first-order kinetic adsorption fitting curve yielded a correlation coefficient (R^2^) of 0.71, indicating poor correlation and that this equation cannot accurately describe the adsorption reaction. The pseudo-second-order kinetic equation fit the reaction better, with a correlation coefficient of 0.998, indicating excellent linear correlation. The calculated theoretical adsorption amount was 5.00 mg/g, which is very close to the experimentally measured adsorption amount of 4.99 mg/g, indicating that the second-order kinetics equation accurately described the adsorption process. The correlation coefficients obtained through Bangham and Elovich kinetics fitting were 0.74 and 0.75, respectively, indicating poor correlation and the inability of these equations to fit the adsorption process.

### 3.6. Adsorption Isotherm

**Figure 4:**
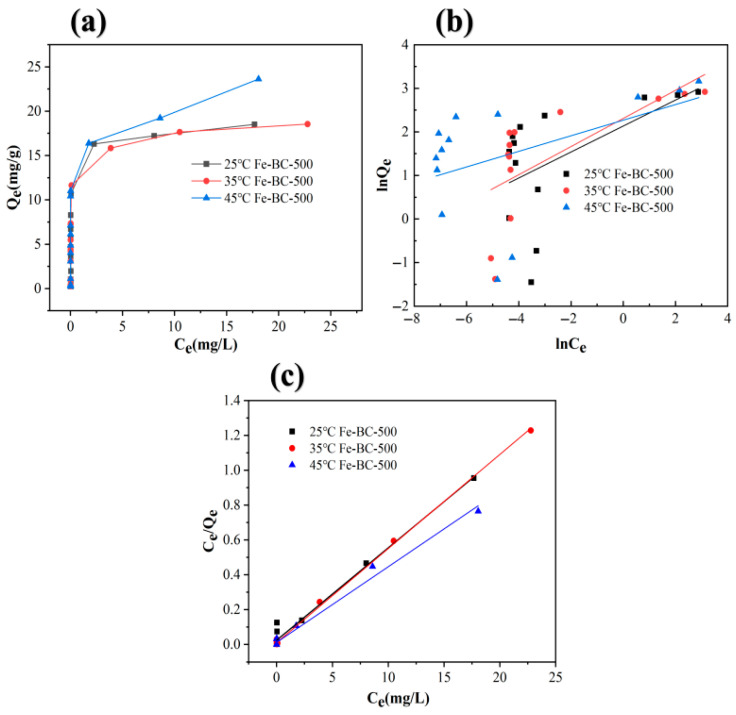
(**a**) Adsorption isotherms of As(V) adsorption on Fe-BC-50; (**b**) Langmuir sorption isotherms; (**c**) Freundlich sorption isotherms.

We added 0.1 g of Fe-BC-500 to 50 mL of As(V) solution with different concentrations (0.5, 1, 2, 4, 6, 8, 10, 12, 15, 20, 30, 40, 50 mg/L), and conducted adsorption experiments at 25, 35, and 45 °C and pH 4. The adsorption isotherms are shown in Figure 4a. At 25 °C, the adsorption capacity of the unmodified biochar (M-BC-500) for As(V) was approximately 3.00 mg/g, while that of Fe-BC-500 was 18.5 mg/g, representing an increase of approximately 84%. As the reaction temperature increased from 25 to 45 °C, the adsorption capacity of Fe-BC-500 increased from 18.51 to 23.6 mg/g, indicating a positive correlation between the adsorption capacity of Fe-BC-500 for As(V) and temperature. This points toward an endothermic reaction. Increased temperatures enhance activation of the adsorbent and accelerate chemical adsorption between adsorbent and adsorbate [66]. Baig et al. [67] used magnetic biochar (MKGB3 and MKGB4) prepared using KGS to sorb As(V) from a solution, with an optimal adsorption capacity of 3.1 mg/g. Ahmad et al. [68] significantly improved the As adsorption performance of cotton stem biochar (CSB) for As through chemical modifications. The CSB modified with nitric acid exhibited the best As adsorption capacity owing to its high specific surface area, developed pore structure, and increased number of surface functional groups. The maximum adsorption capacity of that CSB was 157 µg·g^−1^ (Langmuir model), and the removal rate of As under experimental conditions ranged between 67 and 95%. Benis et al. [69] successfully improved the As(V) adsorption performance of rapeseed straw biomass and biochar in water via electrochemical modification. The maximum adsorption capacities of rapeseed straw biomass and biochar after modification increased to 766 and 922 µg·g^−1^, respectively. Compared with these modified biochar adsorbents, Fe-BC-500 has a higher adsorption capacity for As(V) and certain environmental remediation advantages.

The Langmuir and Freundlich equations were used to fit the adsorption isotherm data. The fitting results are shown in Figure 4b,c. The fitting parameters are listed in Table 2. The correlation coefficients for the data fitted by the Freundlich equation at 25, 35, and 45 °C were 0.246, 0.422, and 0.144, respectively, indicating poor correlation and inability to accurately describe the adsorption process. The 1/*n* parameters of the Freundlich equation were 0.289, 0.322, and 0.178 at 25, 35, and 45 °C, respectively, indicating the strong adsorption capacity of Fe-BC-500 for As(V). The Langmuir equation showed higher correlation coefficients (0.981, 0.998, and 0.987 at 25, 35, and 45 °C, respectively) than the Freundlich equation. The maximum adsorption capacities were 18.51, 18.54, and 23.62 mg/g, respectively. The Langmuir equation well described the adsorption process of As(V) on Fe-BC-500, indicating monolayer adsorption (chemical adsorption).

### 3.7. Adsorption Thermodynamics

**Figure 5:**
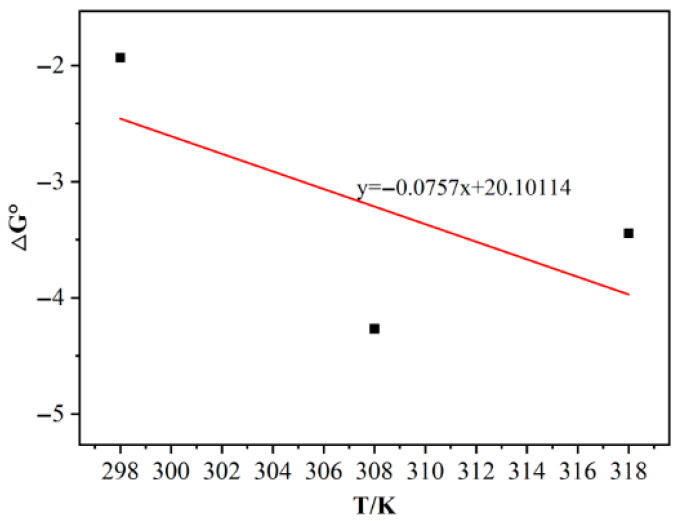
Adsorption thermodynamic of As(V) adsorption on Fe-BC-500.

According to adsorption isotherm analysis, the Langmuir equation more consistently described the adsorption process of As(V) on Fe-BC-500. We thus substituted the Langmuir equation constant K_L_ for the adsorption of As(V) on Fe-BC-500 at different adsorption temperatures into the adsorption thermodynamic equation and calculated the Gibbs free energy △G by combining the Van’t Hoff equation with the Gibbs Helmholtz equation. We then linearly regressed △G against T. The slope −△S and intercept △H are shown in Figure 5. The relevant parameters are listed in Table 3.

The free energies △G of adsorption at 25, 35, and 45 °C were −1.931, −4.266, and −3.445 kJ/mol, respectively, indicating an endothermic reaction. The adsorption intercept ΔH was 20.1011 kJ/mol, indicating a spontaneous chemical adsorption process. ΔS was 0.0757, indicating an entropy-increasing process.

### 3.8. Analysis of Adsorption Mechanisms

#### 3.8.1. FTIR Analysis(FTIR Chemical Bond Analysis)

**Figure 6:**
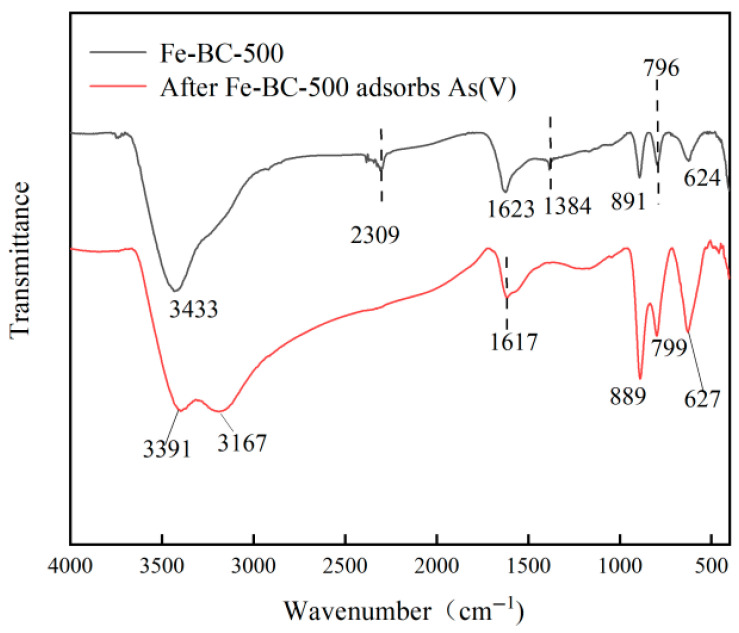
FTIR spectra of Fe-BC-500 before and after As(V) adsorption.

Figure 6 shows infrared spectra of Fe-BC-500 before and after adsorption of As(V). The broad peak at wave number 3433 cm^−1^ is the stretching vibration peak of hydroxyl (OH), which split into dual peaks of 3391 and 3167 cm^−1^ after adsorption. Moreover, the peak shape changed, indicating that hydroxyl participates in adsorption and undergoes ligand exchange, hydrogen bonding formation, or coordination reactions, leading to a change in the O-H vibration mode. The 2309 cm^−1^ peak is characteristic of background CO_2_. The 1623 cm^−1^ peak is the stretching vibration peak of carbonyl C=O and unsaturated alkyl C=C, which shifted to 1617 cm^−1^ after adsorption, indicating interaction between C=O or C=C and As(V). That is, either the lone pair of electrons on the oxygen atom of carbonyl C=O or the delocalized electrons in conjugated large π-bonds coordinated with As. The peaks at 891, 796, and 624 cm^−1^ are the anti-symmetric and symmetric stretching vibration peaks of Fe-O. After adsorption, the peak positions exhibited a small blue shift and intensity change. At the same time, peaks matching the As-O vibration appeared at 889 and 799 cm^−1^, indicating the formation of AsO_4_^3−^. The Fe sites coordinated with As(V) to form Fe–As bonds. We thus infer that the adsorption of As(V) on Fe-BC-500 involves multiple synergistic mechanisms. The hydroxyl groups on the surface of the biochar or iron-based sites underwent ligand exchange with the oxygen-containing acid groups H_2_AsO_4_^−^ and HAsO_4_^2−^, and −OH was replaced by AsO_4_^3−^, further enhancing the Fe-As coordination effect. This is consistent with the splitting and deformation of the hydroxyl region peak. In aqueous solutions, As(V) mainly exists in the form of anions. The Fe-BC-500 surface, especially the Fe-based oxide sites, is positively charged at a suitable pH and adsorbs anionic As(V) through electrostatic attraction, providing an initial driving force for coordination complexation [70]. The FTIR results further showed a significant change in peak shapes before and after adsorption, which is consistent with the results of Zhao et al. [71], indicating that As can be adsorbed on the surface of Fe-BC-500 through ion exchange. The shift in peak positions not only confirms the existence of Fe-O-As internal coordination but also indicates the substitution between surface carboxyl groups and active sites when As(V) exists as anions, leading to changes in the vibration mode.

#### 3.8.2. X-Ray Diffraction Analysis(XRD Crystal Structure Characterisation)

The XRD data were subjected to phase analysis using Jade, in which the standard spectrum file used for comparative analysis was sourced from the PDF ICDD database. Origin was used to draw the XRD spectra, and the phases of the XRD peaks were labeled based on Jade analysis results.

**Figure 7:**
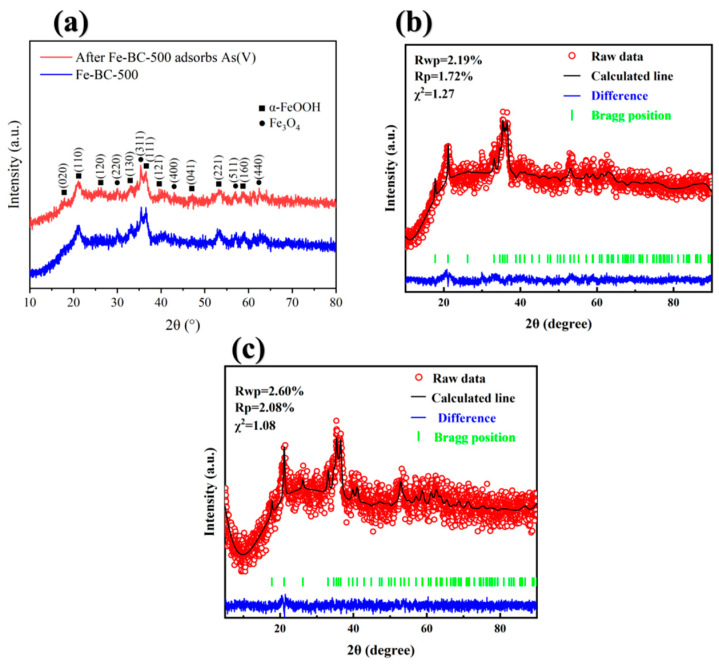
(**a**) X-ray diffraction spectra of Fe-BC-500 before and after As(V) adsorption; (**b**) X-ray diffraction refinement spectrum of Fe-BC-500 before As(V) adsorption; (**c**) X-ray diffraction refinement spectrum of Fe-BC-500 after As(V) adsorption.

The XRD results for Fe-BC-500 before and after adsorption of As(V) are shown in Figure 7a, and X-ray diffraction refinement spectra of Fe-BC-500 before and after As(V) adsorption are shown in Figure 7b,c. We compared the XRD patterns before and after adsorption with the diffraction peaks of standard card Fe_3_O_4_ (97-009-8088) and FeO(OH) (97-003-7156) and could clearly separate the characteristic diffraction peaks of Fe_3_O_4_ and FeO(OH).

We observed peaks at diffraction angles (2θ) of approximately 21.1°, 33.1°, 36.4°, 39.8°, and 53.1°, corresponding to the (110), (130), (111), (121), and (221) crystal planes of goethite (α-FeOOH), respectively, as shown in the standard pattern PDF#01-073-6522. Needle iron ore belongs to the orthorhombic crystal system with the space group Pbnm(62). In addition, the diffraction peaks observed at 29.9°, 35.4°, 43.3°, 57.1°, and 62.7° correspond to the (220), (101), (400), (511), and (440) crystal planes of magnetite (Fe_3_O_4_), respectively, as shown in the standard spectrum PDF#01-088-0866. Magnetite is a cubic crystal system with space group Fd-3m(227). The XRD results indicate that Fe-BC-500 was composed of two phases: Goethite and magnetite. After adsorption of As(V), the XRD pattern did not show significant changes compared with that before adsorption, and the positions and intensities of the diffraction peaks remained essentially the same. These results indicate that adsorption of As(V) did not cause any changes in the crystal structure of the material. The XRD peaks before and after adsorption were both wide, indicating small grain size and relatively low crystallinity. The preparation Fe-BC-500 and its NO_3_^−^ adsorption properties revealed characteristic peaks of Fe_2_O_3_ (JCPDS card No. 33-0664) at 35.54°, 39.41°, and 50.05°, further confirming the successful loading of Fe onto the biochar. This aligns with the characteristic peak observed at 35.4° in the Fe-BC-500 sample after As adsorption [72,73]. No new crystals were generated during the adsorption of As(V) on Fe-BC-500. The widths of the diffraction peaks before and after adsorption were essentially the same, indicating no significant change in grain size after adsorption. These results indicate that As(V) is mainly physically adsorbed on Fe-BC-500, and that the microstructure of the material remained stable and undamaged during the adsorption process. The adsorption process is thus likely dominated by surface interactions via electrostatic interactions or van der Waals forces, without significant chemical bonding or crystal phase transitions. The peak intensity weakened after adsorption, possibly because of chemical reactions with the functional groups on the Fe-BC-500 surface.

#### 3.8.3. Scanning Electron Microscopy(Surface Morphology Observation by SEM)

**Figure 8:**
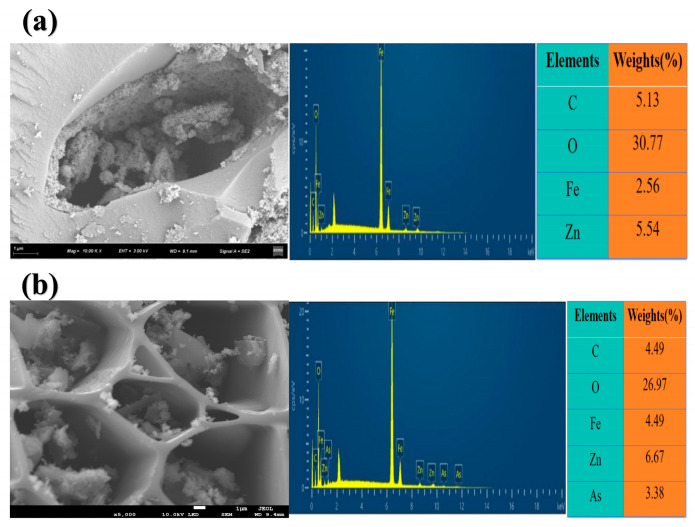
SEM spectra and EDS surface scan elemental results of Fe-BC-500 (**a**) before and (**b**) after adsorption of As(V) on Fe-BC-500.

As shown in Figure 8, Fe-BC-500 exhibited a porous and loose microstructure before adsorption of As(V), with an uneven distribution of voids and obvious void and particle accumulation on the surface. This porous structure provides physical adsorption sites for the adsorption of As(V). Compared with those before adsorption, the pores of Fe-BC-500 after adsorption of As(V) were filled, surface particle accumulation was denser, and the “porosity” of some pores was reduced. This is because As(V) ions were adsorbed by the material and adhered to the pore surface or filled the pores, resulting in “densification” of the microstructure. These patterns are similar to those found by Zhang et al. [72], who observed the appearance of many granular sediments and an increasingly uneven surface bulge distribution of iron-biochar upon combination with other substances. This phenomenon demonstrates the adsorption effect of As(V) on the materials. As(V) adheres to the surfaces of materials through physical and chemical interactions, changing the original porous structure. The main elements on the surface of the samples were analyzed using Scanning Electron Microscope-Energy Dispersive Spectroscopy, as shown in Figure 8a,b. The results showed that a large amount of substances was loaded onto the surface and pores of Fe-BC-500 before and after adsorption of As(V). The corresponding energy spectrum showed that Fe-BC-500 contained C, O, Zn, and Fe (in the order Fe > O > C) before adsorption of As(V), indicating the presence of Fe on its surface. The presence of Zn was due to the addition of ZnCl solution during modification. After adsorption of As(V), Fe-BC-500 contained C, O, Fe, Zn, and As. Comparison of the SEM images after adsorption of As(V) indicated that the original rough structure on the surface of Fe-BC-500 was covered by new and dispersed granular materials. EDS surface-scanning elemental analysis indicated strong As signals in these new material regions, whose spatial distribution was highly consistent with the Fe signal. This result directly confirms that As(V) was effectively adsorbed and enriched on the iron oxide sites and within the pores on the Fe-BC-500 surface.

## 4. Conclusions

In this study, we systematically investigated the adsorption behavior of As(V) on Fe-BC-500, focusing on the effects of pH, initial As(V) concentrations, and co-existing anions. To this end, we combined adsorption kinetics, isotherms, and thermodynamics, as well as XRD, FTIR, SEM, and other methods. The results showed that Fe-BC-500 had the highest adsorption capacity for aqueous As(V) at pH 4 and initial As(V) concentrations of 2, 10, and 20 mg/L. However, pH did not significantly affect adsorption at an As(V) concentration of 2 mg/L, while it had a significant effect on adsorption at initial As(V) concentrations of 10 and 20 mg/L. The influence of co-existing anions on adsorption was relatively small and in the order PO_4_^3−^ > SO_4_^2−^ > NO_3_^−^. Moreover, NO_3_^−^ did not have a significant effect on As(V) adsorption on Fe-BC-500. Adsorption dynamic studies showed that the adsorption rate was relatively fast during the initial 9 h and reached equilibrium within 60 h, a pattern that was well fit by the second-order kinetic model (R^2^ = 0.998). Isothermal adsorption at 25, 35, and 45 °C was highly consistent with the Langmuir model, with R^2^ values of 0.981, 0.998, and 0.987, respectively. The maximum adsorption capacity qmax was 18.51, 18.54, and 23.62 mg/g, respectively, indicating monolayer chemical adsorption. XRD, FTIR, and SEM-EDS analyses revealed that the functional groups -C=O, -OH, and -COOH on the surface of Fe-BC-500 were involved in As(V) adsorption. After adsorption, diffraction peaks characteristic of Fe_3_O_4_ and FeO(OH) appeared. However, the crystal spacing did not significantly differ before and after adsorption, indicating no new formation of iron oxide/arsenide crystal phases. Adsorption was mainly based on electrostatic attraction, surface diffusion, and surface complexation, indicating single-layer chemical adsorption. As signals could be detected on the Fe-BC-500 surface after adsorption, confirming effective adsorption of As(V).

## Figures and Tables

**Table 1:** Kinetic parameters of As(V) adsorption on Fe-BC-500.

Kinetic Equation	Rate Constant	Q_e_ (mg/L)
Pseudo first order kinetics	*K*_1_ = 0.02	0.166
Pseudo second order kinetics	*K*_2_ = 2.00	5.00
Bangham kinetics	*K*_3_ = 0.02	1.84
Elovich kinetics	*K*_4_ = 0.12	-

**Table 2:** Isotherm parameters of As(V) adsorption on Fe-BC-500.

	Langmuir Equation			Freundlich Equation		
Temperature	R^2^	K_L_	Qe	K_F_	1/*n*	R^2^
25 °C	0.981	2.18	18.9	8.45	0.298	0.246
35 °C	0.998	5.29	18.5	10.1	0.322	0.422
45 °C	0.987	3.68	23.0	9.67	0.178	0.144

**Table 3:** Adsorption thermodynamic parameters of As(V) adsorption on Fe-BC-500.

T (K)	K_L_ (L/mg)	△G° (kJ/mol)	△S° [kJ/(mol·k)]	△H (kJ/mol)
298	2.18	−1.931	0.0757	20.1011
308	5.29	−4.266		
318	3.68	−3.445		

## Data Availability

The original contributions presented in the study are included in the article, and further inquiries can be directed at the corresponding author.
